# Neurofeedback Training for Cognitive and Motor Function Rehabilitation in Chronic Stroke: Two Case Reports

**DOI:** 10.3389/fneur.2019.00800

**Published:** 2019-07-24

**Authors:** Wenya Nan, Ana Paula Barbosa Dias, Agostinho C. Rosa

**Affiliations:** ^1^Department of Psychology, Shanghai Normal University, Shanghai, China; ^2^Department of Bioengineering, LaSEEB-System and Robotics Institute, Instituto Superior Tecnico, Universidade de Lisboa, Lisbon, Portugal

**Keywords:** alpha, neurofeedback, chronic stroke, rehabilitation, cognition

## Abstract

Stroke is a debilitating neurological condition which usually results in the abnormal electrical brain activity and the impairment of sensation, motor, or cognition functions. In this context, neurofeedback training, i.e., a non-invasive and relatively low cost technique that contributes to neuroplasticity and behavioral performance, might be promising for stroke rehabilitation. We intended to explore neurofeedback training on a 63-year-old male patient and a 77-year-old female patient with chronic stroke. Both of them had suffered from an ischemic stroke for rather long period (more than 3 years) and could not gain further improvement by traditional therapy. The neurofeedback training was designed to enhance alpha activity by 15 sessions distributed over 2 months, for the purpose of overall cognitive improvement and hopefully also motor function improvement for the female patient. We found that the two patients showed alpha enhancement during NFT compared to eyes open baseline within most sessions. Furthermore, both patients reduced their anxiety and depression level. The male patient showed an evolution in speech pattern in terms of naming, sentences completion and verbal fluency, while the female patient improved functionality of the march. These results suggested that alpha neurofeedback training could provide a spectrum of improvements, providing new hope for chronic stroke patients who could not gain further improvements through traditional therapies.

## Introduction

Stroke is a debilitating neurological condition caused by interruptions in the blood supply to the brain. One in six people worldwide will suffer from a stroke throughout their lives. Being the main cause of adult disability, stroke usually results in the changes in electrical brain activity and the impairment of sensation, motor or cognition functions (1). In this context, as a non-invasive and relatively low cost technique that contributes to neuroplasticity, EEG neurofeedback training (NFT) might be a promising tool for stroke rehabilitation. By addressing excesses and deficits in particular EEG activities through real-time inhibition or augmentation training, NFT enables the patient to modulate their neuronal electrical activity and thus on cortical metabolism.

Following NFT, some stroke patients showed modest improvements in cognitive functions such as attention, memory, concentration, reading, and coordination speech (2). For instance, following upper alpha NFT, two single chronic stroke patients with memory deficits showed memory improvement meanwhile cortical “normalization” was found in a stroke patient with pathological brain activation patterns (3). Compared to traditional rehabilitation, thirty sessions of sensorimotor rhythm (SMR, 12–15 Hz) or mid-beta (15–18 Hz) NFT was more effective to improve concentration and visual perception for the patient with hemi-paralysis from stroke within the previous 3 months to 1 year (4). In summary, previous studies have shown the potential of NFT for cognition rehabilitation in stroke survivors.

Ischaemic stroke is a result of blockage of a blood vessel supplying the brain. Attenuation of alpha power may occur following ischaemic stroke (5). Alpha power plays important roles in not only cognitive functions but also physiological conditions. It has been found that alpha power had a relatively strong positive correlation with the regional cerebral blood flow (rCBF) (5). Moreover, alpha power enhancement was associated with improvements in motor functions and activities in daily living (6, 7).

Although NFT has demonstrated benefits on stroke rehabilitation, whether it still works on the chronic stroke patients who had suffered from stroke with long period (above 3 years) and failed to gain further improvement using traditional therapies is not clear yet. Therefore, we applied NFT on two patients with chronic stroke, in order to examine whether they were able to increase their alpha activity by NFT and consequently gain improvement in cognition or motor ability.

## Materials and Methods

### Participants

Two patients with chronic stroke were recruited according to the criteria that they were able to understand the tasks, had sense of time and space, and had suffered stroke for more than 6 months. The traditional rehabilitation therapies stopped working for both participants. Written informed consents were obtained from both participants before the experiment. The protocol was in accordance with the Declaration of Helsinki and approved by the Research Ethics Committee of the CHLN and CAML.

Participant A was a 63-year-old man, 80 kg and 1.64 m tall, married and professional active. He had two ischemic strokes in February and November 2013, respectively, due to atheromatous disease of large vessels. A computed tomography (CT) scan revealed cortico-subcortical hypodensity in the left frontal-temporal-operculum region and occlusion of the middle cerebral artery. For his rehabilitation background in the past, he performed physical rehabilitation three times per week in a clinic to treat his right hemiparesis until no further improvement could be obtained. He recovered his gait, but still had difficulties to make functional hand movements. He also had conduction aphasia caused by stroke and was treated with speech therapy, but he stopped it when he had no further improvement. He presented cognitive deficits, had difficulties in pronouncing certain words, but understood all kinds of speech. His dyslipidemia and hypertension were controlled daily with medication. He was followed by medical doctors all the time, with routine appointments. The NFT started in 2016 during which no other intervention was performed.

Participant B was a 77-year-old woman, 75 kg and 1.47 m tall, widowed and retired. In October 2006, she suffered from ischemic stroke, and a CT scan revealed hypodensity in the parieto-temporal region with stenosis of the right vertebral artery. For her rehabilitation background in the past, she performed physical rehabilitation three times per week in a clinic until no further improvements could be achieved. When NFT started in 2016, she presented difficulties in her gait, but she walked independently with crutches. She was with sequelae in the right hemisphere and symptoms of hemiparesis, slightly increased tonus, abolition of postural sensitivity, hemiosthosthesia of the right limbs, decreased right visual acuity and peripheral left facial paresis. She had insulin-treated secondary diabetes post-acute pancreatitis and hypertension, both medicated daily. She was followed by medical doctors all the time, with routine appointments. She had no cognitive deficits. No other treatment was performed during NFT intervention.

### Performance Assessment

In order to examine NFT effects, several assessments were performed before NFT, during NFT, and after NFT as follows. The Hospital Anxiety and Depression Scales (HADS), forward and backward digit span tests were conducted for both participants. HADS was used to assess anxiety and depression, in which seven items were relate to anxiety and another seven items were relate to depression (8). The range of scores for each scale is 0–21, with higher scores indicating a worse condition. Forward and backward digit span test were utilized to assess short term memory and working memory.

We also focused on the most affected functions by stroke, i.e., the speech function in Participant A and motor function in Participant B. Consequently, the following tests were tailored to each participant before NFT, in the middle of NFT, and after NFT. The speech ability of Participant A was assessed by the tests adapted from Mini-Mental State Examination (MMSE) that is extensively used in clinical and research setting (9) and extended with many test related to expression from the Brief Aphasia Evaluation (BAE) that can detect minimum verbal performance in patients with aphasia (10). We tested naming (including image and color identification), sentences completion, verbal fluency, reading and repeating, sentence writing, and copying.

The motor ability of Participant B was measured through the Berg Balance Scale (11), Ten Meter Walk Test (12), and Timed Up and Go (13). More specifically, in order to evaluate the balance improvement and reduce the test time due to the patient condition, five tasks (Task 1, 2, 4, 6, 7) related to balance were chosen based on the Berg Balance Scale. Ten Meter Walk Test and Timed Up and Go were employed to evaluate the walking speed.

### NFT Experiment

For comfort and attendance of participants, the experiment was conducted in their homes without the occurrence of distractions and noise. Participants were asked to remain sitting comfortably in front of a computer. To avoid artifacts, they were instructed not to speak and to remain as still as possible during NFT. There was no other therapy involved during NFT intervention.

The training location was determined according to both NFT target and participant condition. Participant A was done at Oz in order to minimize the interference of his involuntary and frequent eye movements. For Participant B, the training location was Cz for cognition and motor function enhancement. The two reference electrodes were placed on the left and right mastoids and the ground electrode was placed over the nasion.

The EEG signals were amplified by an EEG amplifier (Compact 823, Meditron, Electomedicina Ltda, SP, Brazil) and recorded by the software Somnium (Cognitron, SP, Brazil) at a sampling frequency of 250 Hz. The signals were filtered with an analog band-pass filter from 0.1 to 70 Hz in the amplifier and a digital band-pass filter from 4 to 30 Hz. The electrode impedances were maintained below 5 kΩ.

Each participant performed 15 sessions totally with 2 sessions per week. When the participants were not available due to their time schedule, the session would be postponed. As a result, both participants completed 15 sessions within 2 months. Each session consisted of 5 training blocks, and each block had 8 90-s trials with a 5-s interval in-between. Thus, the total training time was 1 h per session. Before and after each session, 1-min EEG baseline with eyes open (EO) and eyes closed resting condition was recorded, respectively.

Considering the large individual difference in the alpha band, individual alpha band (IAB) was adopted for the training location of each patient (14). IAB was defined by the EEG spectrum crossings between the eyes open and eyes closed resting conditions. The IAB of Participant A was between 9.7 and 11.3 and his peak alpha frequency was 10.8 Hz. For Participant B, IAB ranged from 6.5 Hz to 9.4 Hz and her peak alpha frequency was 8 Hz.

The relative amplitudes were calculated by the following equation where the Low was the lower boundary of the frequency band and the High was the higher boundary of the frequency band, and the *X*(*k*) is the frequency amplitude spectrum calculated by fast Fourier transformation (FFT) every 125 ms with a 2-s data window.

$$Relative\;amplitude\;=\;\frac{\frac{\sum_{k=Low}^{High}X(k)}{High-Low}}{\frac{\sum_{k=4}^{30}X(k)}{30-4}}$$

A sphere and a cube displayed on a computer screen were utilized for real-time visual feedback. The radius of the sphere reflected the feedback parameter in real time, and if this value reached a threshold (Goal 1) the sphere color changed. This sphere was made of several slices and the more slices it had, the smoother it looked. The cube height was related to the period of time for which Goal 1 kept being achieved continuously. If Goal 1 was being achieved continuously for more than a predefined period of time (2 s), Goal 2 was accomplished and the cube rose up until Goal 1 stopped being achieved. Then the cube started falling slowly until it reached the bottom or Goal 2 was achieved again (15). Therefore, the participants were instructed to try different mental strategies to increase the sphere size or keep the cube as high as possible. No explicit mental strategy was given, and they were told to be guided by the feedback process instead.

The threshold in the first training block of each session was set to slightly lower than the alpha in the EO resting baseline measured before each session. For the remaining blocks, the threshold would be increased by 0.1 in the next block if the percent time above threshold was above 60%.

## Results

### EEG Result

**Figure 1:**
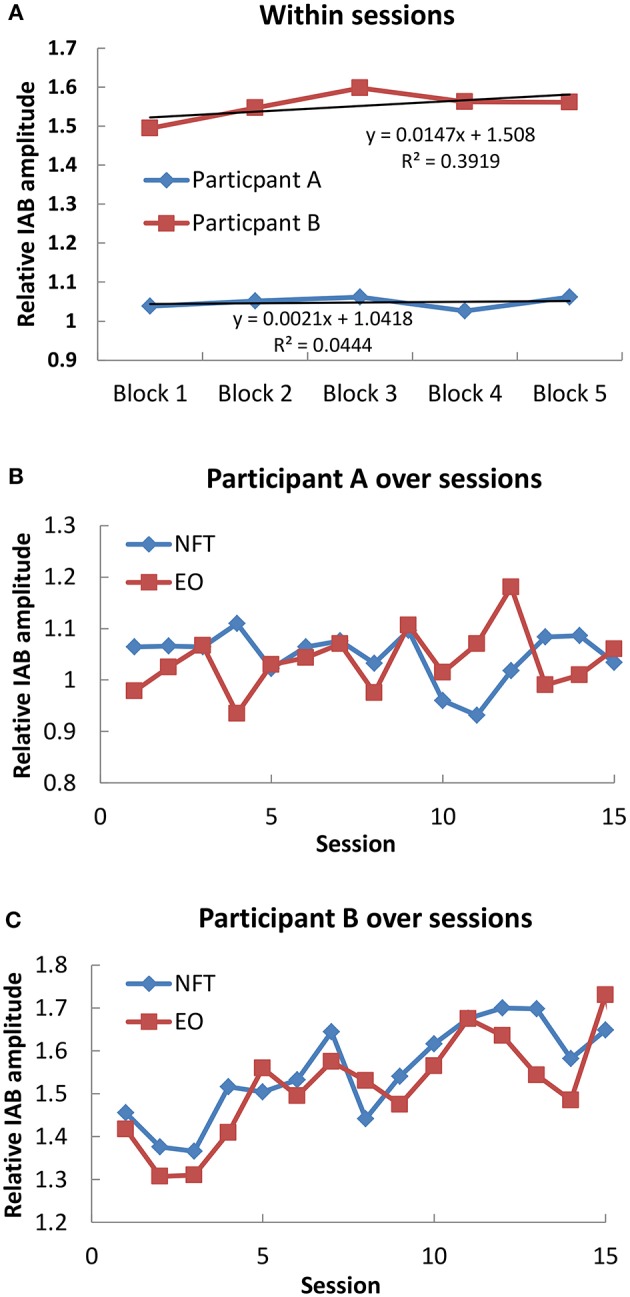
Mean IAB over time. **(A)** Within sessions; **(B)** Participant A over sessions; **(C)** Participant B over sessions.

In each NFT block and resting baseline, the relative IAB amplitude was calculated for further analysis. We firstly examined the IAB change within sessions. As shown in Figure 1A, Participant A only showed a slight increase trend over 5 NFT blocks, while Participant B presented a more obvious increasing trend over 5 NFT blocks.

Secondly, we examined the IAB change across sessions. The average IAB amplitude across 5 NFT blocks within sessions were taken as session activity, and the average IAB amplitude between pre and post NFT baseline was regarded as EO baseline on each training day. For Participant A as shown in Figure 1B, no obvious increase trend over whole NFT procedure could be found in either NFT sessions (*r* = −0.255, *p* = 0.359, 2 tailed) or EO baseline (*r* = 0.303, *p* = 0.272, 2 tailed). Nonetheless, he could increase his IAB during NFT compared to EO baseline in 9 sessions, accounting for 60% of total sessions. Regarding Participant B across whole training process in Figure 1C, she could learn to increase her IAB in both NFT sessions (*r* = 0.798, *p* = 0.001, 2 tailed) and EO baseline (*r* = 0.71, *p* = 0.003). Furthermore, she could elevate IAB amplitude during NFT compared to EO baseline in 80% of total sessions.

### Performance Result

For digit span test, Participant A did not show any change in either forward or backward digit span over NFT procedure, whereas Participant B increased one digit after NFT. The anxiety and depression scores of the two participants reduced over NFT process (Table 1).

**Table 1:** Anxiety and depression score.

**Scale**	**Participant A**			**Participant B**		
	**Before**	**In the Middle**	**End**	**Before**	**In the Middle**	**End**
Anxiety	10	10	9	11	9	8
Depression	12	11	10	10	9	8

As expected, Participant A had some difficulties in carrying out tests due to his cognitive deficits. His speech assessment results were shown in Table 2. It can be seen that there was a slight evolution in the naming including images identification and color identification, sentences completion, and verbal fluency. The remaining tests did not show any evolution, but there was no setback in any performed test.

**Table 2:** Speech assessment result of Participant A.

**Tests**		**Before**	**After 5 sessions**	**After 11 sessions**	**End**
Images identification (score 0–10)		8	9	10	10
Color identification (score 0–5)		3	4	4	4
Sentences completion (score 0–5)		2	3	3	3
Verbal fluency	A	5	5	5	6
	B (score 0–21)	14	19	21	21
	C (score 0–1)	1	1	1	1
Reading and repeating (score 0, 3, 5)		3	3	3	3
Sentence writing (score 0–1)		0	0	0	0
Sentence copying (score 0–1)		1	1	1	1

For Participant B, in the Berg Balance Scale test related to balance, improved performance in Task 1 and Task 6 was observed over NFT sessions, whereas other tasks remained constant over time. Additionally, she improved walking speed in 10 Meter Walk Test and Time Up and Go (Table 3). It should be noted that this patient had a fall a week before the last assessment, which influenced her motor ability score at the end of NFT.

**Table 3:** Motor ability result of Participant B.

**Tests**		**Before**	**After 5 sessions**	**After 11 sessions**	**End**
Berg Balance Scale	Task 1	2	2	3	3
	Task 2	3	3	3	3
	Task 4	3	3	3	3
	Task 6	3	4	4	4
	Task 7	3	3	3	3
10 Meter Walk Test		8.9 s	8.4 s	8.1 s	8.3 s
Time Up and Go		14.2 s	13.9 s	13.5 s	13.5 s

## Discussion

Both participants finished the NFT without experiencing obvious adverse effects. For the across session effects, it was found that Participant B could learn to increase her IAB amplitude over sessions, which also led to enhancement in EO baseline. The same is no longer true for Participant A who had no obvious increase trend in either NFT or EO baseline across sessions. Similarly, regarding the within session effects, Participant A did not show obvious increase trend in IAB over 5 NFT blocks, whereas Participant B was much better than Participant A. Nevertheless, Participant A could learn to elevate his IAB in 9 NFT sessions compared to EO baseline, indicating that he could show higher IAB activity in NFT blocks than resting state within short term but the maintenance of such increase was not easy over longer periods. Similarly, Participant B also learnt to increase her IAB in most NFT sessions when compared with EO baseline. These results suggested that NFT yielded some plastic changes in brain activity, but such change had inter-individual difference.

The inter-individual differences in neurofeedback learning have also been found in previous work, regardless of the NFT protocol and subject population (16–23). With regard to stroke patients, Kober et al. (24) which investigated the NFT effects on cognitive functions also found that not all patients could learn to enhance alpha amplitude within and across NFT sessions. In healthy population the ability to enhance alpha has been found to be positively related to resting alpha activity, i.e., the individual who has higher resting alpha activity is more likely to gain successful enhancement of alpha by NFT (25). Such relation might be generalized to the patients with chronic stroke, as Participant B who had much higher EO alpha than Participant A (1.42 in Participant B and 0.98 in Participant A before the first session) showed much larger alpha increase within sessions and across sessions.

Following alpha NFT, the two participants have slight improvement in their emotional well-being. The levels of anxiety and depression dropped to a more relaxed behavior. For the adapted tests, a slight evolution in speech performance was found in Participant A who had symptoms of conduction aphasia. Likewise, improved speed of the march was observed in Participant B. The NFT sessions were performed at patients' home, and there was no change of their exercise routine. Furthermore, only NFT was performed without other therapy during NFT intervention. Thus, it is speculated that the improvement was only due to NFT. Furthermore, it has been shown that in the course of recovery within 6 months after stroke, alpha increase is associated with improvement in motor performance and activities of daily living (6). Our results suggested that with the help of NFT, the chronic stroke patients were able to enhance their alpha activity, and achieve improvement on rehabilitation, even though the time from stroke onset was rather long (at least above 3 years) and the patients cannot be further improved using traditional therapy.

Prior case reports also showed positive effects of NFT on stroke rehabilitation but with different NFT protocols. For instance, Rozelle and Budzynski (26) employed beta1/theta NFT with the purpose of beta1 enhancement and theta reduction on a male stroke patient. After NFT, the patient reduced his slow-wave activity, depression, anxiety, and tinnitus. Additionally, he improved his speech fluency, word finding, balance and coordination, attention, and concentration. Mroczkowska et al. (27) conducted SMR/theta NFT in order to enhance SMR and decrease theta on a female stroke patient with aphasia, and obtained positive results in terms of concentration, visual perception, categorizing, as well as the regulation of affect and reduction in the aphasia symptoms. While the present study suggested that alpha NFT also could contribute on stroke rehabilitation, especially in the patient with chronic stroke who could not gain further improvement from traditional therapy.

## Conclusion

In conclusion, alpha NFT appeared to have induced some brain plasticity in chronic stroke patients, which was associated with improvement of emotional state, cognitive, and motor functions. It should be noted that even a slight improvement is promising, since the patients had no further improvement using traditional therapy. Our result suggested the potential of NFT for chronic stroke rehabilitation.

## Data Availability

The datasets generated for this study are available on request to the corresponding author.

## Ethics Statement

All subjects gave written informed consent in accordance with the Declaration of Helsinki. The protocol was approved by the Ethics Committee of the CHLN and CAML.

## Author Contributions

WN, AD, and AR designed research. AD conducted the experiment and analyzed data. WN and AD wrote the manuscript. AR and WN supervised, revised, and gave the final approval of the manuscript. All authors read and approved the manuscript.

### Conflict of Interest Statement

The authors declare that the research was conducted in the absence of any commercial or financial relationships that could be construed as a potential conflict of interest.

## References

[1] Murphy TH Corbett D. Plasticity during stroke recovery: from synapse to behaviour. Nat Rev Neurosci. (2009) 10:861–72. 10.1038/nrn2735 19888284

[2] Renton T Tibbles A Topolovec-Vranic J. Neurofeedback as a form of cognitive rehabilitation therapy following stroke: a systematic review. PLoS ONE. (2017) 12:e0177290. 10.1371/journal.pone.0177290 28510578 PMC5433697

[3] Kober SE Schweiger D Reichert JL Neuper C Wood G. Upper alpha based neurofeedback training in chronic stroke: brain plasticity processes and cognitive effects. Appl Psychophysiol Biofeedback. (2017) 42:69–83. 10.1007/s10484-017-9353-5 28197747 PMC5344963

[4] Cho HY Kim K Lee B Jung J. The effect of neurofeedback on a brain wave and visual perception in stroke: a randomized control trial. J Phys Ther Sci. (2015) 27:673–6. 10.1589/jpts.27.673 25931705 PMC4395689

[5] Finnigan S van Putten MJAM. EEG in ischaemic stroke: quantitative EEG can uniquely inform (sub-)acute prognoses and clinical management. Clin Neurophysiol. (2013) 124:10–9. 10.1016/j.clinph.2012.07.003 22858178

[6] Giaquinto S Cobianchi A Macera F Nolfe G. EEG recordings in the course of recovery from stroke. Stroke. (1994) 25:2204–9. 7974546 10.1161/01.str.25.11.2204

[7] Sauseng P Klimesch W Gerloff C Hummel FC. Spontaneous locally restricted EEG alpha activity determines cortical excitability in the motor cortex. Neuropsychologia. (2009) 47:284–8. 10.1016/j.neuropsychologia.2008.07.021 18722393

[8] Zigmond AS Snaith RP. The hospital anxiety and depression scale. Acta Psychiatr Scand. (1983) 67:361–70. 10.1111/j.1600-0447.1983.tb09716.x 6880820

[9] Folstein M Folstein S McHugh P. “Mini-mental state”. A practical method for grading the cognitive state of patients for the clinician. J Psychiatr Res. (1975) 12:189–98. 1202204 10.1016/0022-3956(75)90026-6

[10] Vigliecca NS Peñalva MC Molina SC Voos JA. Brief aphasia evaluation (minimum verbal performance): concurrent and conceptual validity study in patients with unilateral cerebral lesions. Brain Injury. (2011) 25:394–400. 10.3109/02699052.2011.556106 21314276

[11] Berg KO Wood-Dauphinee SL Williams JI Maki B. Measuring balance in the elderly: validation of an instrument. Can J Public Health. (1992) 83(Suppl 2):S7–11. 1468055

[12] Bohannon RW. Comfortable and maximum walking speed of adults aged 20-79 years: reference values and determinants. Age Ageing. (1997) 26:15–9. 9143432 10.1093/ageing/26.1.15

[13] Ng SS Hui-Chan CW. The timed up and go test: its reliability and association with lower-limb impairments and locomotor capacities in people with chronic stroke. Arch Phys Med Rehabil. (2005) 86:1641–7. 10.1016/j.apmr.2005.01.011 16084820

[14] Klimesch W. EEG alpha and theta oscillations reflect cognitive and memory performance: a review and analysis. Brain Res Rev. (1999) 29:169-95. 10.1016/S0165-0173(98)00056-3 10209231

[15] Nan W Rodrigues JP Ma J Qu X Wan F Mak PI. Individual alpha neurofeedback training effect on short term memory. Int J Psychophysiol. (2012) 86:83–7. 10.1016/j.ijpsycho.2012.07.182 22864258

[16] Hanslmayr S Sauseng P Doppelmayr M Schabus M Klimesch W. Increasing individual upper alpha power by neurofeedback improves cognitive performance in human subjects. Appl Psychophysiol Biofeedback. (2005) 30:1–10. 10.1007/s10484-005-2169-8 15889581

[17] Weber E Koberl A Frank S Doppelmayr M. Predicting successful learning of SMR neurofeedback in healthy participants: methodological considerations. Appl Psychophysiol Biofeedback. (2011) 36:37–45. 10.1007/s10484-010-9142-x 21053066

[18] Zoefel B Huster RJ Herrmann CS. Neurofeedback training of the upper alpha frequency band in EEG improves cognitive performance. Neuroimage. (2011) 54:1427–31. 10.1016/j.neuroimage.2010.08.078 20850552

[19] Enriquez-Geppert S Huster RJ Scharfenort R Mokom ZN Zimmermann J Herrmann CS. Modulation of frontal-midline theta by neurofeedback. Biol Psychol. (2014) 95:59–69. 10.1016/j.biopsycho.2013.02.019 23499994

[20] Reichert JL Kober SE Neuper C Wood G. Resting-state sensorimotor rhythm (SMR) power predicts the ability to up-regulate SMR in an EEG-instrumental conditioning paradigm. Clin Neurophysiol. (2015) 126:2068–77. 10.1016/j.clinph.2014.09.032 25743268

[21] Baumeister S Wolf I Holz N Boecker-Schlier R Adamo N Holtmann M Neurofeedback training effects on inhibitory brain activation in ADHD: a matter of learning? Neuroscience. (2016) 2016:25 10.1016/j.neuroscience.2016.09.025 27659116

[22] Hsueh JJ Chen TS Chen JJ Shaw FZ. Neurofeedback training of EEG alpha rhythm enhances episodic and working memory. Hum Brain Mapp. (2016) 37:2662–75. 10.1002/hbm.23201 27038114 PMC6867560

[23] Quaedflieg CW Smulders FT Meyer T Peeters F Merckelbach H Smeets T. The validity of individual frontal alpha asymmetry EEG neurofeedback. Soc Cogn Affect Neurosci. (2016) 11:33–43. 10.1093/scan/nsv090 26163671 PMC4692315

[24] Kober SE Schweiger D Witte M Reichert JL Grieshofer P Neuper C. Specific effects of EEG based neurofeedback training on memory functions in post-stroke victims. J NeuroEng Rehabil. (2015) 12:1. 10.1186/s12984-015-0105-6 26625906 PMC4666277

[25] Wan F Nan W Vai MI Rose A. Resting alpha activity predicts learning ability in alpha neurofeedback. Front Hum Neurosci. (2014) 8:500. 10.3389/fnhum.2014.00500 25071528 PMC4095646

[26] Rozelle GR Budzynski TH. Neurotherapy for stroke rehabilitation: a single case study. Biofeedback Self-regul. (1995) 20:211–28. 10.1007/bf01474514 7495916

[27] Mroczkowska D Białkowska J Rakowska A Neurofeedback as supportive therapy after stroke. Case report Postȩpy Psychiatrii i Neurologii. (2014) 23:190–201. 10.1016/j.pin.2014.09.002

